# Complex mental health difficulties in primary care: a scoping review with thematic synthesis

**DOI:** 10.3399/BJGPO.2024.0223

**Published:** 2025-12-19

**Authors:** Kritica Dwivedi, Vyv Huddy, Phillip Oliver, Chris Burton

**Affiliations:** 1 Division of Population Health, The University of Sheffield, Sheffield, United Kingdom; 2 Clinical Psychology, The University of Sheffield, Sheffield, United Kingdom; 3 Division of Population Health, The University of Sheffield, Sheffield, Sheffield, United Kingdom; 4 Division of Population Health, The University of Sheffield, Sheffield, United Kingdom

**Keywords:** mental health, family medicine, systematic review, general practice

## Abstract

**Background:**

Complex mental health difficulties (CMHD) is an umbrella term for long-term problems with emotions and relationships, including personality disorders (PD), persistent depression, and consequences of trauma. People with CMHD often fall between NHS services that focus on either common mental disorders (anxiety, depression) or psychosis, leaving GPs as their main source of support.

**Aim:**

To understand what is known about primary care for CMHD, from both GP and patient perspectives.

**Design & setting:**

We conducted a scoping review of GP and patient experiences of CMHD in primary care in UK, Europe, Australasia, and North America.

**Method:**

We searched MEDLINE, PsycInfo, and Embase for eligible studies between January 2002 and October 2023. Titles and full texts were screened by two reviewers. Thematic synthesis of qualitative studies and narrative synthesis of quantitative studies were undertaken.

**Results:**

We screened 2209 papers and 33 met inclusion criteria. The following three key themes were found: the challenge of recognising CMHD; the work of caring for people with CMHD; and patient priorities. GPs recognised CMHD through complexity of diagnoses, of psychosocial issues, and of healthcare use. However, they were ambivalent about diagnosis and lacked the resources to make or discuss diagnoses. Working with people with CMHD involved responsibility work, relationship work, and emotional work, under pressured conditions. Patient priorities included addressing stigma, reducing fragmentation, and receiving relationship-focused care.

**Conclusion:**

This scoping review delineates the very real challenges people with CMHD and their GPs face in providing care. It helps set an agenda for work to address gaps in provision and improve outcomes.

## How this fits in

This is the first systematic scoping review focusing on the experiences of GPs and patients with complex mental health difficulties (CMHD) in primary care. The challenges reported by GPs agree with previously reported experiences of other healthcare professionals working with CMHD in primary care. Patients value continuity and trusting relationships, but feel the increasingly fragmented care GPs can provide is at times counterproductive. The evidence suggests that GPs need appropriate resources and interprofessional collaboration with specialists to provide important stability, validation, and support for patients with CMHD.

## Introduction

Complex mental health difficulties (CMHD) is a generic term to describe difficulties more persistent or disruptive than common mental disorders but which do not meet current definitions of severe mental illness such as psychosis or bipolar disorder.^1^ Sometimes also referred to as complex emotional needs, they are characterised by repeated episodes of anxiety and depression, with long-term unpredictable changes in mood and difficulties in relationships.^2–5^ The term overlaps with diagnostic entities including personality disorders (PD), persistent depression (dysthymia), comorbid substance misuse, and the consequences of trauma. CMHD was proposed in a 2018 Mind consensus statement, which recognised ‘personality disorder’ as a contested and often stigmatising diagnosis.^1^

CMHD are common, with the global prevalence of PD alone estimated to be between 6% and 12%.^6–8^ This increases further in different healthcare settings, with studies reporting rates of 20% in emergency departments,^9,10^ 24% in primary care attenders,^11^ 40%–50% in psychiatry outpatients,^12^ and 55%–70% in referrals to primary care counselling services such as Improving Access to Psychological Therapies (IAPT).^13,14^ However,<1% prevalence of diagnostic coding of PD is reported in primary-care electronic healthcare records,^15,16^ suggesting poor recognition in general practice.

CMHD are associated with self-harm and suicide,^17–19^ medical and mental health comorbidities,^15,20^ and lower life expectancy.^20,21^ They disproportionately affect people in the least affluent communities, for several reasons including early exposure to adversity,^22,23^ poverty, and structural inequities.^24^ People with CMHD frequently experience forms of social exclusion^25^ and difficulties recognising and trusting the intentions of others.^26,27^ NHS services are often organised for uncomplicated common mental health disorders and severe mental illness, with CMHD falling in the gaps,^28^ leading to care that is episodic and crisis-related.^29,30^ This is opposed to the continuity of care they value, with a trusted health professional.^31,32^

A recent task force highlighted the importance of an integrated role for primary care in managing complexity in mental health.^33^ While there is a moderately large literature of patient and clinician experience of specialist and community mental health care for CMHD,^2–4,18,34,35^ much less is known about primary care services and CMHD.

## Method

### Identifying the research question

The following two key questions were identified:

1. What is known about people with CMHDs' experiences and attitudes towards primary care involvement in their care?
2. What is known about GPs’ experiences and attitudes towards providing care for people with CMHD?

### Identifying relevant studies

The project was registered at Open Science Framework (DOI 10.17605/OSF.IO/TDGBS).^36^ Searches were conducted in three databases: MEDLINE, PsycInfo, and Embase, between January 2002 and October 2022. An updated search was run in October 2023. Two complementary search strategies were used, combining concepts of CMHD, general practice, community health care, and qualitative research. The detailed search criteria are described in Supplementary Material 1. Additional forward and backward searching from citations and references were conducted from key papers.

### Study selection

Screening of titles and abstracts was conducted independently by two reviewers (CB and VH). Screening of full texts for inclusion was conducted by the same reviewers with 20% double rated, and uncertainties and disagreements resolved by discussion.

Studies were considered eligible for inclusion if they:

- involved adults with CMHD identified using a range of generic terms (complex near mental, emotional, or psych*), or specific diagnoses such as PDs; persistent or recurrent depression or dysthymia;
- examined patient or clinician experiences, views, and attitudes using semi-structured interviews or focus groups, or reported demographic or clinical characteristics of patients;
- focused on a general practice (family medicine or primary care) setting in the UK, Europe, Australasia, or North America;
- were written in English and published in a peer-reviewed journal.

Studies limited to children or adolescents, solely focusing on older adults, or in specialist mental health services (hospital or community) were excluded. Case studies, reviews without explicit search and sifting, and survey data were also excluded.

### Charting the data

Data on basic descriptive characteristics, for example, study types, setting, and aims, was independently extracted by two reviewers (CB and VH) using an Excel-based form. A data-charting form was developed collaboratively and reviewed regularly to reflect the data found. Two reviewers (KD and CB or VH) independently extracted data for thematic synthesis. Charts were compared between reviewers and accuracy of extraction and suitability of charting features was iteratively monitored and updated. Quantitative data reporting prevalence, mental health comorbidities, prescribing, and healthcare usage was extracted (KD).

### Collating, summarising, and reporting the results

A descriptive analysis of key study characteristics was reported. Qualitative data were analysed using thematic synthesis, with both explicit or implicit themes of papers considered. Theme labels, data, and commentary were matched to similar or linked themes to produce a cohesive synthesis. As there were insufficient studies reporting comparable data for meta-analysis, quantitative data were reported as a narrative synthesis.

## Results

### Study selection

**Figure 1.**
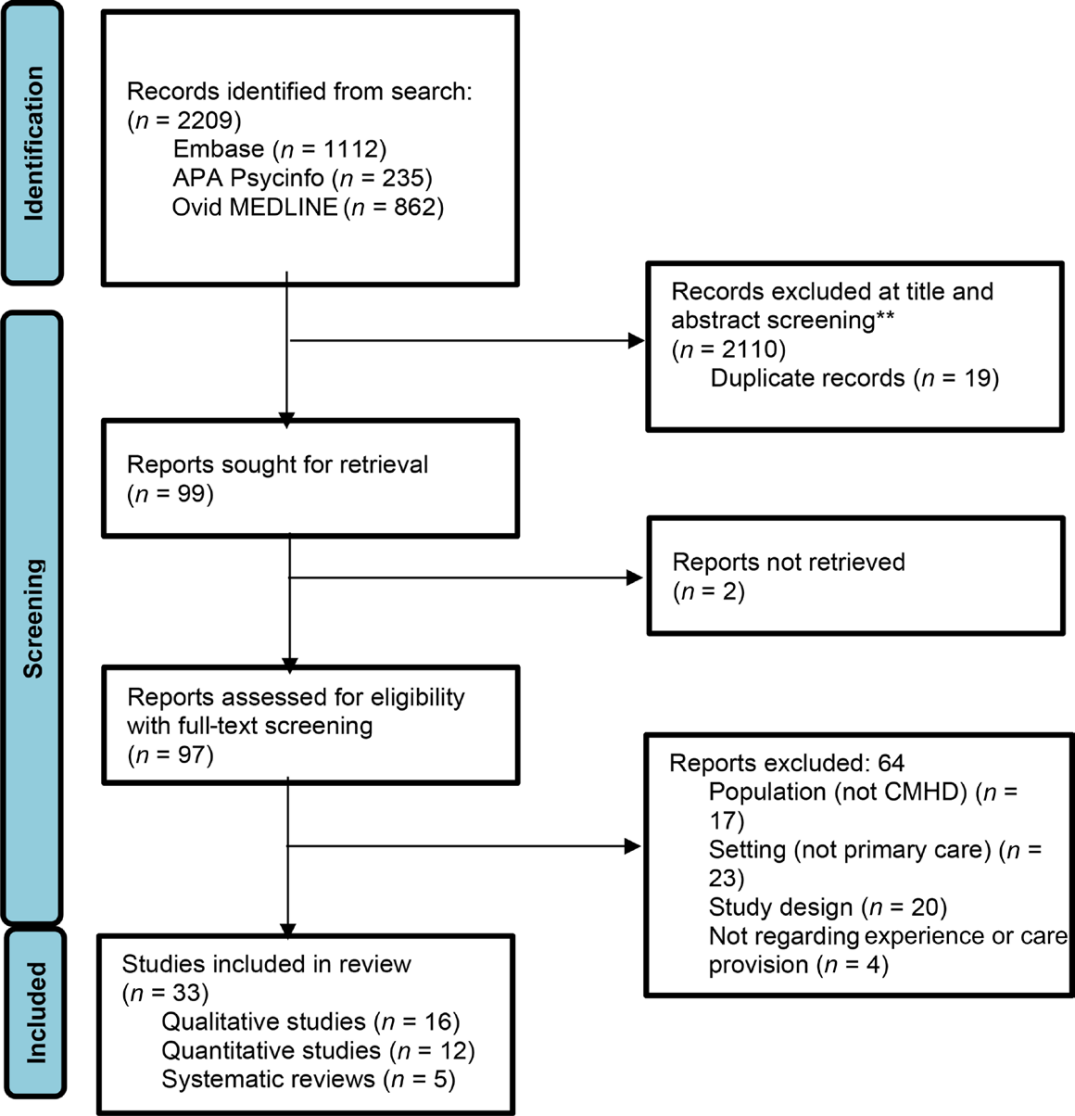
Preferred Reporting Items for Systematic reviews and Meta-Analyses (PRISMA) flow diagram for study selection, adapted from the PRISMA 2020 statement.^62^ APA = American Psychological Association. CMHD = complex mental health difficulties

The screening process is summarised in a Preferred Reporting Items for Systematic reviews and Meta-Analyses (PRISMA) diagram (Figure 1). In total, 2209 initial reports were identified from the search, from which 99 abstracts were selected for full-text review. This was completed for 97 (as two could not be retrieved) and resulted in 33 eligible papers. This comprised 28 primary papers and five systematic reviews. Searching references from the published reviews did not identify any new papers for inclusion.

### Summary of studies

The eligible primary papers included 16 qualitative and 12 quantitative studies. The most common diagnosis of interest was PD (18 studies, with six focusing on borderline personality disorder [BPD] specifically), followed by recurrent depression (two studies), with the remaining eight studies focusing on a combination of general or complex mental health conditions rather than specific diagnoses. Studies were most commonly set in the UK (16 papers), followed by Australia (four papers), Canada (two papers), and one each from other European Countries (Denmark, Finland, the Netherlands, Spain, and Sweden) and US. Supplementary Tables S1 and S2 summarise the included qualitative and quantitative studies, respectively.

### Quantitative findings

The quantitative studies comprised five cohort,^19,20,37–39^ five cross-sectional,^11,13,15,16,40^ and two case-control studies.^17,41^ Four studies used healthcare records or databases for analysis.

### Prevalence

Five studies reported an estimated prevalence for at least one form of PD ^11,13,15,16,40^, but used different populations and ascertainment methods. These are summarised in Table 1, which highlights the contrast between prevalence in treatment settings and low rates of coding in GP records.

**Table 1.** Summary of reported prevalence for personality disorder (PD) and borderline PD in different settings

First author and year	Country	Study setting	Population	Method	Prevalence
Personality disorder					
Sundquist 2017^15^	Sweden	Primary care records	Primary care registry	Presence of ICD-10 code	0.3%
Moran 2000^11^	UK	Primary care	Consecutive attenders to GP surgery	informant-based interview for standardised assessment of personality	23.8%
Jones 2006^13^	UK	Primary care referrals	GP referrals to psychology service for CBT	Self-report measure: Millon Clinical Multiaxial Inventory (MCMI-III)	56.4%
Borderline personality disorder					
Aragonès 2003^16^	Spain	Primary care records	Primary care records of Catalan Health Institute	Presence of BPD (code F60.3/ICD-10)	0.017%
Sansone 2011^40^	US	Outpatients	Consecutive sample of internal medicine outpatients	Self-report survey: Personality Diagnostic Questionnaire-4 (PDQ-4) and Self-Harm Inventory (SHI)	10.2% (PDQ-4), 12.1% (SHI)

BPD = borderline personality disorder. CBT = cognitive behavioural therapy. ICD = International Classification of Diseases

### Mental health comorbidities, prescribing, and healthcare usage

Comorbid mental health conditions were reported in six studies featuring patients with PD. ^11,13,15,16, 39,41^ Three studies reported different aspects of prescribing in PDs, two of which specifically examined antipsychotic prescribing and found a rate of 25%–34%.^16,39^ Three studies reported healthcare usage or costs^16^,^37,40^ but with varied study populations. These findings are summarised in Table 2.

**Table 2.** Summary of reported mental health comorbidities, prescribing rates, and healthcare usage

First author and year	Country	Study setting	Population	Mental health comorbidity	Prescribing rates	Healthcare usage and costs
Moran 2000^11^	UK	Primary care attenders	Consecutive attenders to GP surgery with PD	62%≥2 PD diagnoses		
Rendu 2002^37^	UK	Primary care	Consecutive attenders to GP surgery with PD			PD were not independently associated with increased costs
Aragonès 2003^16^	Spain	Primary care records	Patients with BPD ICD-10 code	26.3% affective disorders	25.2% antipsychotics, 37.6% antidepressants, 37.4% anxiolytics	Visited the primary care doctor twice as often as the general adult population in Catalonia (8.1 versus 4.4 annual visits)
Jones 2006^13^	UK	Primary care referrals for CBT	GP referrals to psychology service for CBT with PD	30.9%≥2 PD diagnoses		
Sansone 2011^40^	US	Outpatients	Consecutive sample of internal medicine outpatients with BPD			Statistically significant: more primary care physicians are seen by patients with BPD compared with no BPD over 5 years (2.85 versus 1.88)
Patel 2015^41^	UK	Primary care	Frequent GP attenders	31% depression, 25% dysthymia, 28% any anxiety diagnosis		
Sundquist 2017^15^	Sweden	Primary care records	Patients with PD ICD-10 code	63% depression, 67% anxiety	80.1% antidepressants, 71.7% anxiolytics	
Hardoon 2022^39^	UK	Primary care records	Patients with PD Read code	75% depression, 51% anxiety	34% antipsychotics	

BPD = borderline personality disorder. CBT = cognitive behavioural therapy. ICD = International Classification of Diseases. PD = personality disorder

In terms of interpretation, these studies were so heterogenous in their study setting and populations, methods used to define PD or other CMHD, and their reported outcomes, it was not possible to formally combine them. Rather they were used as a context within which the qualitative data could be analysed.

### Thematic synthesis

**Figure 2.**
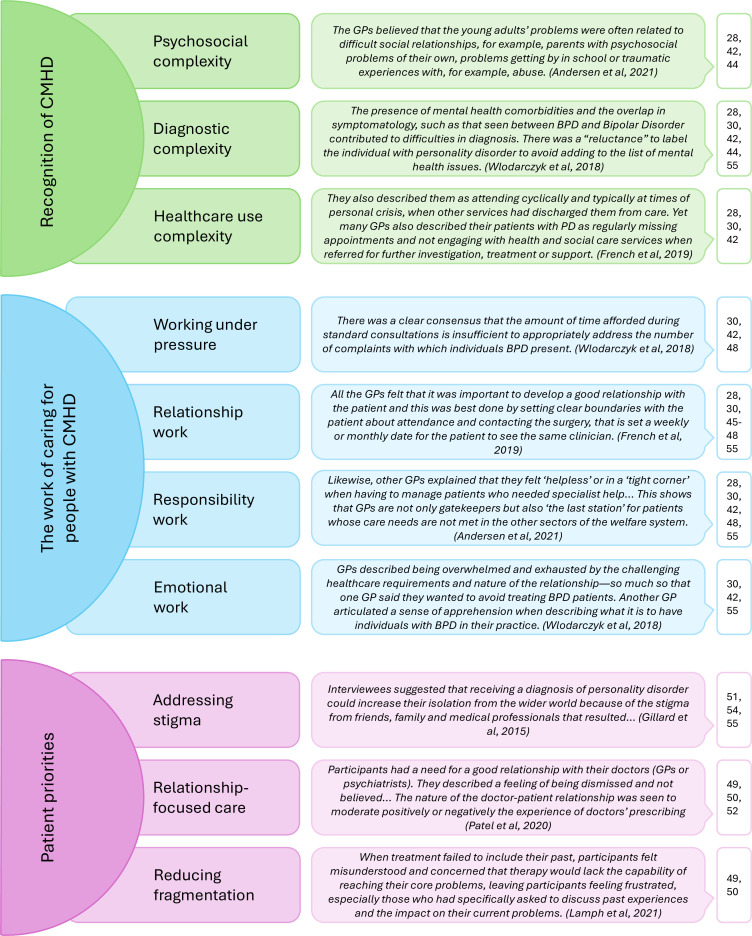
Summary infographic of themes and sub-themes generated from thematic synthesis, with example excerpts from studies

Of the 16 qualitative studies, nine reported healthcare professional experiences^28,30,42–48^ and six reported patient experiences.^49–54^ The patient experience studies included other community services and had relatively little content directly relevant to primary care. One study reported from focus groups of varied participants.^55^ We identified the following three key themes: the recognition of CMHD in primary care; the work of caring for people with CMHD; and patient priorities. These are summarised in Figure 2.

#### Recognition of complex mental health difficulties

GPs recognised this group of patients in various ways, which we have grouped as psychosocial complexity, diagnostic complexity, and healthcare use complexity.

##### Psychosocial complexity

Psychosocial complexity was mentioned in three studies.^28,42,44^ GPs highlighted previous traumatic experiences and difficult social relationships, describing people with PD '*as living chaotic lives, with a history of substantial adversity across their lives*'.^28,42^ Psychosocial issues and mental disorders were seen as entangled. However, GPs also noted that this complexity had to be '*reduced to a clear psychiatric diagnosis*' to access specialist input.^42^

##### Diagnostic complexity

Multiple complaints or diagnoses were typical in patient presentations.^28,30,44^ ^,55^ GPs highlighted how the combination of multiple symptoms and comorbidities with a perceived lack of knowledge or psychiatric expertise prevented them from making a diagnosis, even when they had strong clinical impressions.^30,42^ Even when GPs had strong clinical impressions, they did not see the act of making a diagnosis as within their skills or remit.^30,42^ Instead, GPs relied more on a *'gut feeling*' or '*clinical instinct'*. This was accompanied by a reluctance to add another, possibly stigmatising, mental diagnostic label such as PD.^30^

##### Healthcare use complexity

GPs reported people with PD to be *'frequent attenders who were often unaware of the amount of practice time they required compared with other patients'*.^28^ They described patients presenting in crisis .^28,30,42^ Furthermore, multiple studies mentioned that patients would often not attend or cancel appointments,^28,30,42^ which made it difficult for GPs *'to track patients’ health over the longer term, and to assess their level of risk'*.^28^ One consequence of non-attendance in general practice was that diagnostic assessment outside of a crisis became more difficult.^30^ This was compounded by non-attendance at specialist services, which resulted in discharge back to the GP.^42^

### The work of caring for people with CMHD

Multiple studies reported how managing CMHD in primary care was challenging work for GPs. This included working under pressure but also the labour involved, which we have categorised as relationship work, responsibility work, and emotional work.

#### Working under pressure

Three studies highlighted time pressures faced by GPs when managing people with CMHD,^30,42,48^ and how this prevented them from adequately addressing the healthcare needs of this group. One study reported *'a clear consensus that the amount of time afforded during standard consultations is insufficient'*.^30^

#### Relationship work

While GPs recognised the importance of therapeutic relationships with patients with PD,^28^ they found these relationships to be demanding. Studies reported on the negative impressions held by GPs of people with PDs^28,30^ and the impact this could have on doctor–patient contacts.^45,48^ GPs reported consultations with patients with PD to be '*unpredictable'* and '*challenging*'.^28^ When GPs were able to collaborate with psychiatrists or psychologists, they felt reassured and supported.^30,46^ Relationship work was hindered by poor continuity of care, both in practices and from specialist services.^48,55^

#### Responsibility work

Multiple studies highlighted how GPs were unable to access specialist advice,^28,48,55^ leaving them *'with full responsibility'* and *'little choice but to improvise their own management plans*'.^28^ GPs commented on *'referrals being knocked back and patients falling in the gaps between services'*, with referrals rejected as they were considered not ‘*risky*’ enough for community mental health teams, but too high risk for primary care mental health services.^28^ Existing treatments were perceived by GPs as inappropriately short term, inflexible, or too focused on crisis management^28,42,48,55^ and the need for *'a service that can "hold" and manage long-term risk'* was highlighted.^28^

#### Emotional work

The emotional work of holding people with CMHD in primary care was also highlighted in multiple studies.^30,42,55^ Studies reported GPs as feeling '*helpless*', '*overwhelmed and exhausted*', or '*powerless to effect long-term improvement'*, particularly when managing people they felt required specialist help.^30,42^ In most instances, GPs felt unsupported when managing the high emotional and healthcare needs of people with CMHD, although specialist supervision did help, when available.^46^

### Patient priorities

Few studies focused on patient experiences of primary care in relation to their CMHD. Instead, studies focused on the experiences of receiving a diagnosis of PD, living with PD, and treatment in a specialist setting. Nonetheless, studies identified the importance to patients of addressing stigma, relationship-focused care, and reducing fragmentation.

#### Addressing stigma

People with CMHD described negative attitudes that contributed to stigma including ideas about untreatability^51^ and attribution of difficulties in engagement to the person even when psychopathology is recognised.^55^ Diagnosis and the label of PD had mixed effects. For some patients a diagnosis of PD gave them valuable knowledge and legitimacy, while for others it exposed them to stigma and subsequent isolation, including from their GP.^51,54^

#### Relationship-focused care

Patients expressed a need for a good relationship with their doctors. Where patients felt they were able to see the GP’s *'human side'* and were listened to, this positively contributed to the doctor–patient relationship and prescribing outcome.^50^ However, they also experienced doctors whom they described as too *'rushed, scripted, and uncaring*'.^50^ One study reported people with CMHD avoiding discussing their mental health with their GP unless for specific medication or physical health reasons.^52^ Some described feeling dismissed when trying to access help, and having to bring a family member to be believed by their GP.^50^

#### Reducing fragmentation

Mirroring the GP experiences, patients also spoke of inflexible and fragmented specialist care, with lack of continuity of care and collaboration between different professionals, which may have impacted appointment attendance.^49,50^ Brief psychological interventions from IAPT were seen as inflexible, shallow, and mechanistic.^49^ Therapies other than CBT were rarely offered, and patients felt misunderstood when treatments failed to include their past.^49^

## Discussion

### Summary

GPs recognised CMHD in a variety of ways; however, they felt they were left with full responsibility for providing care for this group but without the capacity. There was a perception that they do not have the tools or resources to address the problems of diagnosis, treatment, and support, exacerbated by workload pressures and lack of interprofessional collaboration with specialists. Patients found that fragmented and transactional care from services could be counterproductive.

### Strengths and limitations

A strength of our review was the broad inclusion criteria. Given the lack of diagnostic coding in GP records, this may better reflect the primary care population.^15,16^ Our use of the term 'complex mental health difficulties' was also influenced by local patient and public involvement, and reflects national policy.^56^ The review protocol was pre-registered and employed a rigorous methodology, with double screening of all titles and abstracts, and iterative discussion between reviewers about emerging concepts for thematic synthesis. The main limitation is that studies focused predominantly on PD, particularly BPD, and other complex mental health problems may have been under-represented in the data. No studies examined comorbid post-traumatic stress disorder (PTSD), and there was a relative lack of patient experiences in primary care, which identifies potential areas for future research.

### Comparison with existing literature

To our knowledge, this is the first systematic scoping review focusing on the experiences of GPs and patients of CMHD in primary care. Previous reviews have focused on specialist settings,^2^ experiences of recovery,^34^ or on BPD specifically.^18,57^

Our themes of GP experiences agreed with experiences of other primary care healthcare professionals reported in the literature.^47,52,58^ Our findings highlighted exclusion from services reported by both patients and GPs, despite the Royal College of Psychiatrists’ position against exclusion.^59^ Our patient findings also aligned well with previous systematic reviews conducted in specialist settings,^2,18,34,35^ and the Mind consensus statement.^1^

There is increasing recognition that PDs are associated with difficulties with mentalising (reading how other people are thinking) and epistemic trust (accepting information as trustworthy and relevant).^18,27^ These difficulties are more likely to occur in conditions of acute stress,^60^ which are likely during episodes of fragmented care. Patients highlighted feeling misunderstood when treatments did not account for their past experiences, which reflects the increasing recognition of trauma-informed care in managing CMHD.^4^ Experiences of stigma, and the need for trusting therapeutic relationships with continuity, flexibility, and stability were highlighted across all care settings.^2,18,34,35^

### Implications for research and practice

The evidence points to an important role of general practice in the care of people with CMHDs. GPs could provide important stability, validation, and support for people with these conditions, but need to be adequately resourced and supported by specialist services. However, there remain significant gaps in knowledge regarding how to achieve this support. Only two studies identified looked at interventions in primary care, such as psychiatrist-supported small group sessions for GPs^46^ or nurse-led proactive reviews.^52^ Case management was also suggested as a means to improve integration and continuity for this group.^55^

Importantly, there is little discussion in the primary care literature about what is the aim of 'treatment'. Whereas there is often a focus on cure or recovery in designing services, this model may not necessarily hold for people with CMHD, who may require longer-term relationship-based support. Short-term focused interventions or crisis response interventions do not adequately address the holistic needs of this group.^28,49,55^ There is a pressing need to identify and implement a patient-centric approach to aims of treatment.^61^ Given the recent expansion of primary care mental health teams in the UK, it is particularly important that the GP and patient perspective on CMHD is considered when designing new services, or they may miss the opportunity to integrate care for people who need it most.

In conclusion, GPs recognise the complexity in this important group of patients but currently lack the resources, interventions, and specialist support to provide long-term, relationship-based care that patients value. Interprofessional team work between specialist services and primary care is needed to address patient needs, and maximise the value of supportive primary care.

## Data

The dataset relied on in this article is available from the corresponding author on reasonable request.
